# Evolutionary Pathways of the Pandemic Influenza A (H1N1) 2009 in the UK

**DOI:** 10.1371/journal.pone.0023779

**Published:** 2011-08-24

**Authors:** Monica Galiano, Paul-Michael Agapow, Catherine Thompson, Steven Platt, Anthony Underwood, Joanna Ellis, Richard Myers, Jonathan Green, Maria Zambon

**Affiliations:** Centre for Infections, Health Protection Agency, London, United Kingdom; University of Hong Kong, Hong Kong

## Abstract

The emergence of the influenza (H1N1) 2009 virus provided a unique opportunity to study the evolution of a pandemic virus following its introduction into the human population. Virological and clinical surveillance in the UK were comprehensive during the first and second waves of the pandemic in 2009, with extensive laboratory confirmation of infection allowing a detailed sampling of representative circulating viruses. We sequenced the complete coding region of the haemagglutinin (HA) segment of 685 H1N1 pandemic viruses selected without bias during two waves of pandemic in the UK (April-December 2009). Phylogenetic analysis showed that although temporal accumulation of amino acid changes was observed in the HA sequences, the overall diversity was less than that typically seen for seasonal influenza A H1N1 or H3N2. There was co-circulation of multiple variants as characterised by signature amino acid changes in the HA. A specific substitution (S203T) became predominant both in UK and global isolates. No antigenic drift occurred during 2009 as viruses with greater than four-fold reduction in their haemagglutination inhibition (HI) titre (“low reactors”) were detected in a low proportion (3%) and occurred sporadically. Although some limited antigenic divergence in viruses with four-fold reduction in HI titre might be related to the presence of 203T, additional studies are needed to test this hypothesis.

## Introduction

In April 2009, several human cases of swine influenza A/H1N1 were confirmed in the USA and Mexico [1]. The viruses responsible were characterised as a novel influenza A H1N1 with a combination of genes not previously detected in human or swine population [2], [3]. The virus rapidly spread to several countries, leading the World Health Organisation in early June to raise the level of pandemic alert to phase 6, as sustained community-level transmission of the virus was taking place in more than one region of the world [4]. Pandemic influenza (H1N1) 2009 viruses were detected for the first time in the United Kingdom (UK) in late April, with the subsequent epidemics manifesting in two broad waves from April to August and September to December. Mathematical modelling estimated that during the first wave of pandemic 320,000 (range 144,000 – 670,000) persons presented symptomatic illness [5].

Gathering antigenic and genetic information about pandemic virus strains was a key task during the 2009 influenza pandemic. It provided a unique opportunity to study the evolution of a novel virus immediately subsequent to its introduction into the human population, so as to understand the genetic pathways underlying the emergence of viral diversity. Global influenza surveillance focuses on monitoring the antigenic and genetic characteristics of circulating influenza strains to aid formulation, with adjustment if necessary, of the annual influenza vaccine composition. Therefore, datasets of HA sequences used in most studies represent influenza strains with unusual antigenic properties or severe clinical outcomes.

In the present study we focus on the analysis of genetic and antigenic characteristics of a set of pandemic H1N1 (2009) viruses with particular advantages, as they were: a) isolated during the first and second pandemic waves in the UK; b) carefully selected to represent chronology, age, severity and geographical distribution and to avoid antigenic bias; c) analised in the context of phylogenetic relationships nationally and globally; d) linked with antigenic data. We investigated the dynamics of circulation of significant genetic variants and the correlation between genetic diversity and antigenicity during the progression of the pandemic in a single country with distinct epidemic waves, high use of prophylactic antivirals and discrete geographical boundaries.

## Results

### Clinical and virological surveillance and phylogenetic analysis

**Figure 1:**
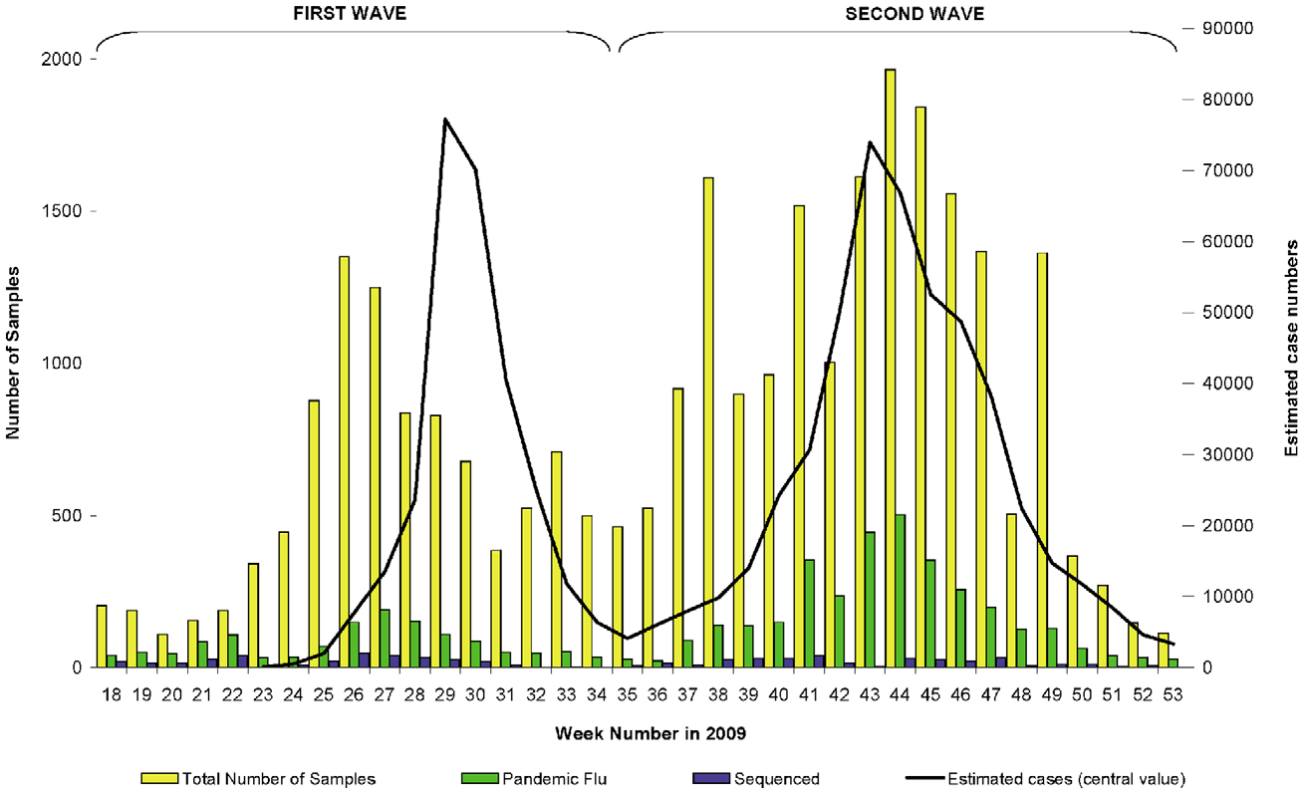
Estimated case numbers & laboratory-confirmed cases of pandemic and seasonal influenza, England, 2009. The estimated number of symptomatic cases with ILI due to pandemic influenza was calculated each week using a statistical model which relies on data from various surveillance systems: the primary care-based QSurveillance scheme, sentinel virological surveillance schemes and data from the National Pandemic Flu Service (NPFS) [61]. Number of pandemic H1N1 viruses selected for sequencing of HA per week is also shown.

The earliest detection of pandemic H1N1 in the UK was on April 27^th^ 2009, at the end of the 2008/09 winter period, in two returning travellers from Mexico, followed by further introductions from travellers from the USA and Mexico. By early June there was sustained community transmission within the UK (weeks 27 to 33 [6]), giving a clear first wave of virus circulation, unlike most European countries which only experienced sporadic or no activity [7]. A second wave of virus circulation occurred during the Northern hemisphere 2009/10 winter (Figure 1). During this period, 28,567 respiratory specimens were tested. A total of 4,673 cases were laboratory confirmed as pandemic H1N1. Peak weeks of virus detection corresponded with peaks of clinical morbidity.

**Figure 2:**
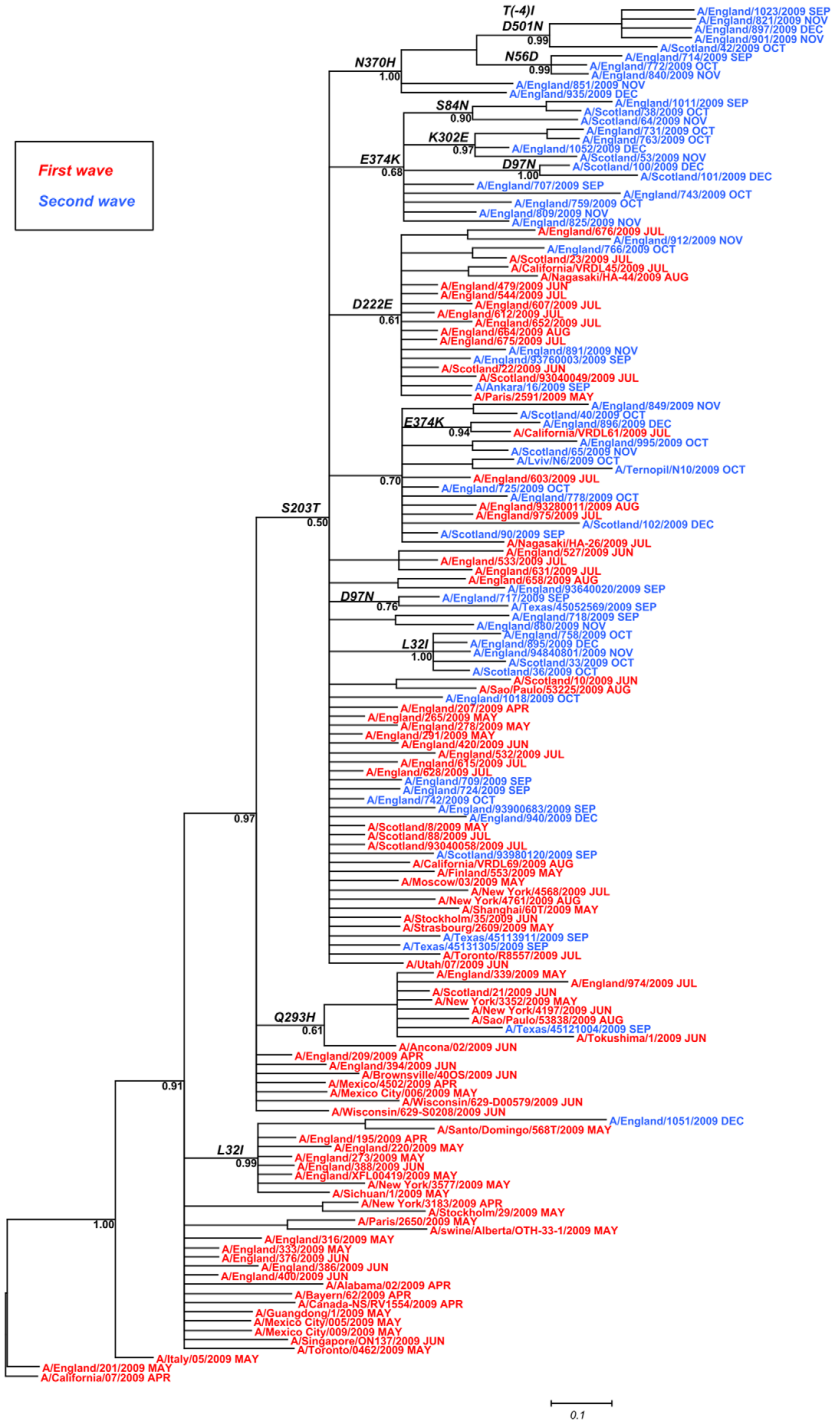
Phylogenetic relationship of full length HA sequences of pandemic influenza viruses isolated in the UK. Viruses from an illustrative subset of 100 were included, as well as additional 50 worldwide viruses, all collected between April and December, 2009. Tips are tagged and coloured according to whether viruses were isolated during the first or second wave. The tree was rooted with A/California/07/2009 as the outgroup. Branch lengths are drawn to scale. Signature amino acid changes are annotated at the nodes of each cluster.

We sought to sequence a representative proportion (10–15%) of laboratory-confirmed cases (Figure 1). Isolates were chosen in order to ensure a uniform coverage geographically, chronologically, clinically and by age group across the period of study (Figure S1). The bias towards younger age groups seen in lab-confirmed cases is coincident with the highest rate of consultation and also with the highest seroincidence in the 5–14 age group during the first and second wave of pandemic in the UK [8], [9]. A total of 685 HA complete coding region sequences from UK viruses (353 from the first wave, 332 from the second wave) plus 475 worldwide HA sequences downloaded from the NCBI Influenza Virus Resource [10] were used to reconstruct a phylogenetic tree by Bayesian methods (Figure S2). For ease of visualisation and analysis, a subset of 100 UK and 50 non-UK viruses were subsequently selected to represent those clusters containing 10 or more sequences (Figure 2).

The HA phylogeny is relatively shallow, with large polytomies and the maximum depth of the tree (from root to furthest tip) being 10 nodes, indicating close evolutionary relationships and subsequent lack of phylogenetic resolution. This is unsurprising, given the short period of time of circulation in humans. The median substitution rate over the period was estimated by Bayesian means at 0.004981 per site per year, although it should be understood that calculating this required estimating divergence times and the estimated substitution rate encompassed a wide range. The majority of viruses, except some early examples located at the root of the tree, group together in a single cluster, characterised by substitution 203T. This cluster includes several minor subclusters which are mostly characterised by other single amino acid substitutions. For these subclusters, some temporal accumulation of substitutions was observed as they include viruses from either the first or the second wave, with bootstrap values of >50%. The coexistence of different clusters indicates co-circulation of lineages. Outside these clusters, most viruses appear on branches with an unresolved topology, consistent with lack of diversity within their HA.

The topography also illustrates that the UK population of viruses is essentially part of the global circulation pool, with several clusters containing UK sequences aligned with geographically distant relatives. Two clusters show a predominance of UK viruses with one US virus, suggesting that a related virus strain from the USA may have seeded those from the UK. However there are some wholly native lineages, including three extended clusters in the second wave that contain only UK sequences. This is consistent with some extended evolution and variation being entirely contained within the UK following chains of transmission, although further sampling may reveal highly related sequences from outside the country. It is important to note that the UK has an extended phase of “containment” with widespread use of prophylactic antivirals during the first weeks of the pandemic between May and June 2009. These analysis indicate that the use of antivirals did not lead to an unusual circulation of variants in the UK.

### Polymorphism and amino acid changes in HA

Genetic analysis of H1N1 (2009) viruses indicated that the HA amino acid sequences were largely similar (sequence similarity of 98.8%–100%) to that of the pandemic prototype and vaccine strain, A/California/07/2009. A maximum difference of 12 substitutions was seen between two viruses from the second wave. Only one virus, A/England/201/2009, isolated on 2^nd^ May 2009 from a returning traveller from Mexico, had an identical HA sequence to that of the vaccine strain; whole genome sequencing confirmed this virus was identical in all gene segments to the vaccine strain (Baillie, Galiano et al, unpublished observations).

**Figure 3:**
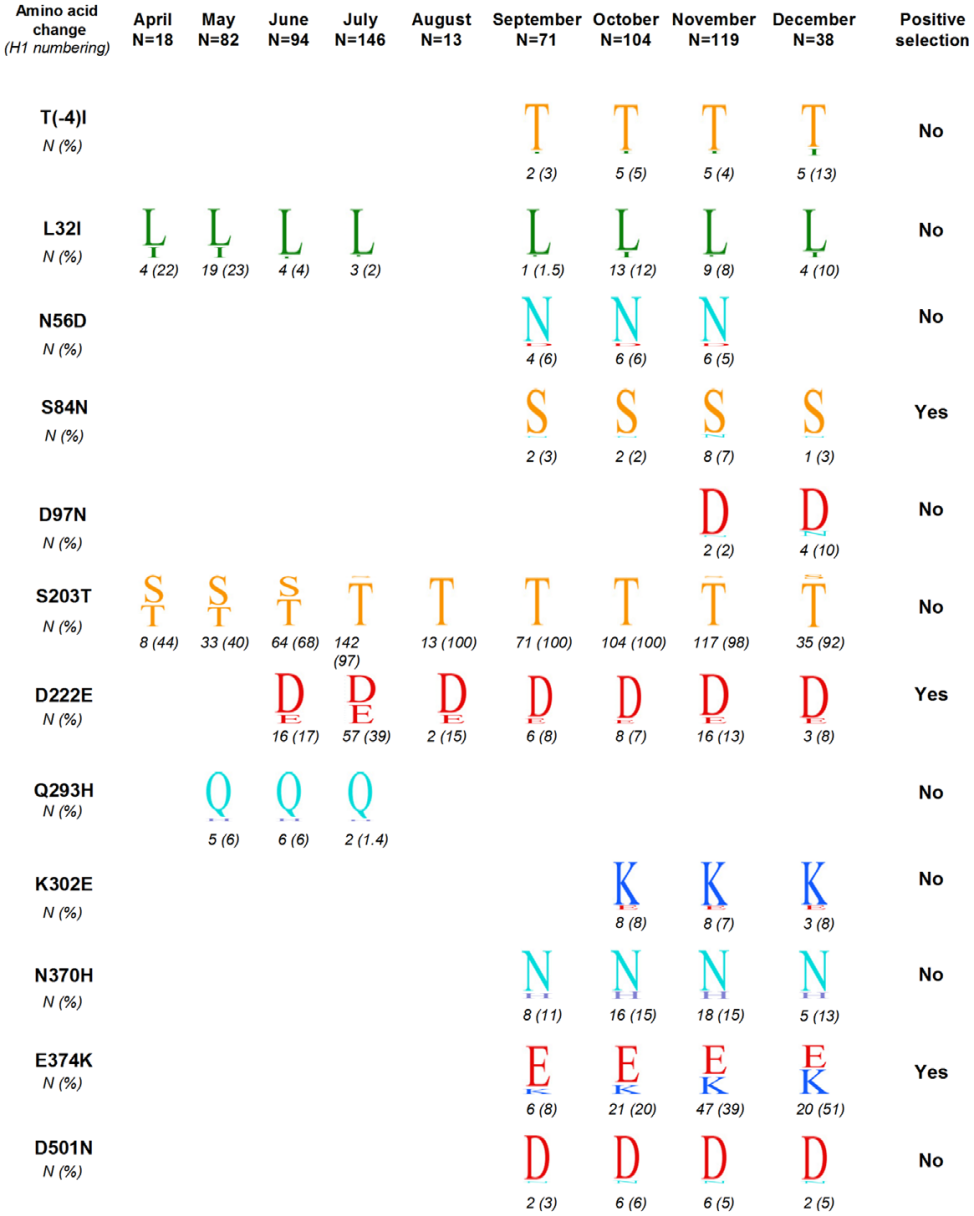
Amino acid changes seen in UK viruses, April – December, 2009. Sites are described using H1 numbering with the HA protein sequence of the vaccine strain A/California/07/2009 used as reference sequence. The logo describes the amino acid changes by displaying the one-letter symbol that codes each amino acid (according to IUPAC – IUB nomenclature) in the proportion seen amongst the analysed viruses. The number of viruses presenting each change is indicated below the logos, with frequencies in brackets.

We analysed the variation in frequency over time of those signature amino acid changes characterising clusters from the phylogenetic trees of Figures 2 and S2. A signature amino acid change is defined as a substitution found exclusively in all sequences within a designated cluster. Figure 3 represents variation month-by-month using the sequence logo format [11]. The most significant observation of this analysis was that substitution S203T gradually increased in frequency from April (44%) to predominate in 100% of viruses from August onwards, a finding consistent with this change also characterising the main branch of the phylogenetic tree (Figure 2). However, a few viruses found later in November-December 2009 showed signs of reversion at this position (203S). Other substitutions appeared with variable frequencies during the pandemic waves. Substitution L32I was seen in up to 23% of viruses from the first wave and 12% from the second wave. Notably, this change characterised two distinct lineages in the tree: a set of first-wave viruses exhibiting 203S and a second-wave group with 203T, suggesting that a new variant exhibiting 203T plus 32I arose during the second wave. In addition, five viruses from November-December which showed 32I had reverted to 203S and clustered with those viruses from the first wave (Figure S2). Change D222E appeared from June onwards in up to 39% and 13% of viruses from the first and second wave respectively. Sporadic occurrence of polymorphism at this position was also detected with change D222G showing in 1–3% of the viruses. Polymorphisms at position 222 have been previously associated with severe clinical outcome [12], [13], [14]. Amongst UK viruses, substitution D222G appeared in 5 of 25 fatal cases, compared to 2 of 668 of non-fatal cases (p value = 0.0001, chi square test). Change D222E was present in 6 of 25 fatal cases vs. 98 of 635 of non-fatal cases (p value = 0.494). In addition, positions 222 and also 203 are located within antigenic site Ca [15], suggesting a possible role for these changes in the generation of antigenic variants.

Most cluster amino acid signatures emerged later during the second wave, perhaps reflecting an accumulation of diversity as is characteristic of influenza viruses. While most second-wave changes were constantly found in around 10–15% of the viruses, substitution E374K notably increased in frequency from 8% in September to 51% in December. Only substitution Q293H was observed in 6% of first-wave viruses but then disappeared after August.

Given the possible relevance of some of these changes on the evolutionary pathways of the H1N1 (2009) viruses, we next sought additional insight by analysing selective pressures acting on the HA deduced protein sequences. To help highlight differences across time, and reduce data to a computationally manageable size, two datasets were constructed using UK sequences collected either during the first or second waves. Figure S3 shows 3 positively selected sites identified in sequences from the first wave and 15 from the second wave, whereas 12 negatively selected sites were found during the first wave and 6 during the second wave. An interesting progression in selection over time is revealed here, with the first wave period featuring mainly negative selection and the second wave mainly positive. Some of negative selection in the first wave could be explained by initial rapid epidemic spread in a largely naïve population coupled with analysis over a short time period. More intensive sampling may detect more transient, weakly deleterious mutations, i.e. those that persist for a small number of generations before being selected to extinction usually because they do not contribute to the virus fitness. The disappearance of these mutations will in turn create the appearance of greater negative selection relation to more long term and less intensive sampling, that will not have observed the mutations in the first instance. Circumstantial evidence for this is given by the occurrence of several amino acid substitutions other than those described in Figure 3 which were sporadically seen in one or a few viruses but did not persist. This would be followed later by increased purifying selection leading to the spread of viruses with advantageous mutations. Three out of 12 sites featuring cluster signature substitutions (84, 222 and 374, Figure 3) were found to be subjected to positive selection mostly during the second wave, suggesting that they may offer some advantage either in terms of adaptive changes to the new host or immune escape. Moreover, position 222 was shown to be consistently subjected to positive selection across both waves, which may reflect its involvement in receptor binding specificity [16].

### Antigenic analysis

**Figure 4:**
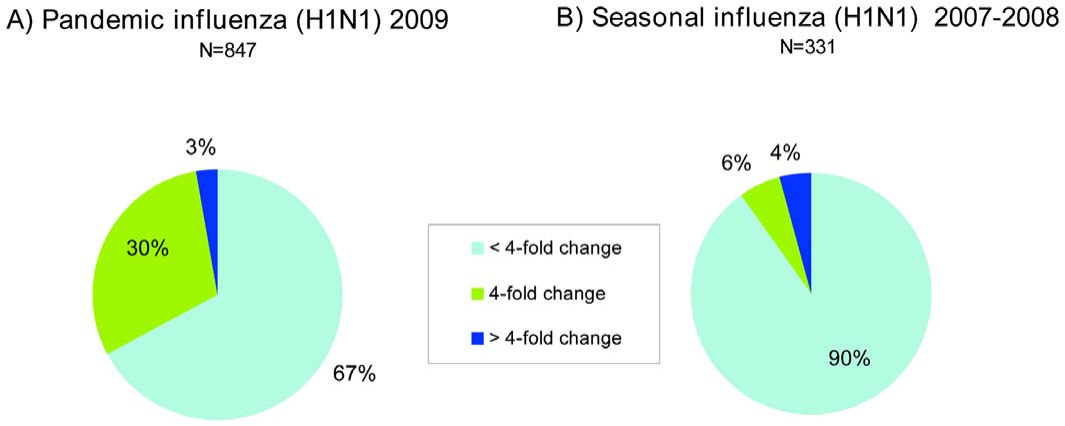
Antigenic reactivities of influenza H1N1 viruses isolated in the UK. Percentage of viruses with different antigenic reactivities as per reduction in HI titres against: a) A/California/07/2009 (pandemic H1N1 2009) antiserum. b) A/Solomon Islands/3/06 (seasonal H1N1) antiserum.

For seasonal influenza, detection of viruses with greater than four-fold reduced reactivity in haemagglutination inhibition (HI) titre compared with a reference prototype strain homologous titre can be an indication of significant antigenic variation. Such viruses are considered as “low reactors”. Given that HI test is a biological assay, the variation which occur within the parameters of the test mean that only four-fold or greater reduction in antigenic reactivity has biological significance. We looked at antigenic characterization data (n = 847) using HI tests with post infection ferret antisera to A/California/07/2009 (vaccine strain), A/England/195/2009 (UK reference strain) and A/Brisbane/59/07 (2008/09 and 2009/10 seasonal H1N1 vaccine strain). The analysis revealed that 97% of pandemic H1N1 (2009) viruses were antigenically similar to A/California/7/2009 (Figure 4a). There were very few (3%) viruses with a greater than four-fold change in reactivity to A/California/07/2009 antiserum (low reactors). When compared to the last influenza season where the seasonal H1N1 influenza subtype had predominated (2007–2008) a similar proportion of “low reactor” viruses was observed (Figure 4b), with no statistically significant difference (p value = 0.287, chi square test). However, the proportion of viruses with four-fold reduced reactivity in the HI titre to the vaccine strain was significantly higher among the pandemic viruses compared to those detected during 2007–2008 (30% vs. 6% for viruses with four-fold reduced reactivity, p value = 0.0001). It is relevant to note that the antigenic analysis of pandemic viruses relied on the use of a single reference ferret antiserum, in contrast to analyses carried out for seasonal influenza, with a panel of reference antisera.

We next looked at those viruses which had both HA sequence and antigenic data available (n = 234). In order to investigate whether any of the signature residues described in Figure 3 could be related to a change in the antigenic reactivity, we cross-matched viruses grouped by their signature changes with their reactivity to A/California/07/2009 antiserum (Table 1). At least 30% of viruses with single substitution 203T or accompanied by 374K, 370H and other minor variants showed four-fold or greater reduced reactivity to A/California/07/2009 antiserum, although only a few of them exhibited greater than four-fold reduction (“low reactors”). Amongst them, viruses with substitutions 56D and 84N were associated with four-fold or greater reduced reactivity to A/California/07/2009 antiserum in 82% and 75% of viruses respectively. It is notable that “low reactor” viruses were distributed throughout the phylogenetic tree (Figure S2).

**Table 1:** Antigenic reactivity of pandemic influenza (H1N1) 2009 viruses from UK (n = 227).

Amino acid signature^a^	N°	Wave	<4-fold^b^	4-fold	>4-fold
			N (%)	N (%)	N (%)
203S, 32I	4	First	3 (75)	1 (25)	0 (0)
293H	1	First	1 (100)	0 (0)	0 (0)
203T only	91	First-Second	61 (67)	27 (30)	3 (3)
**203T, 370H only** c	10	Second	4 (40)	6 (60)	0 (0)
203T, 370H, D501N	8	Second	5 (62.5)	3 (37.5)	0 (0)
**203T, 370H, 56D**	11	Second	2 (18)	7 (64)	2 (18)
203T, 374K only	37	Second	22 (59)	14 (38)	1 (3)
**203T, 374K, 84N**	12	Second	3 (25)	8 (67)	1 (8)
203T, 374K, 97N	7	Second	5 (71)	1 (14)	1 (14)
203T, 374K, 302E	12	Second	8 (67)	4 (33)	0 (0)
**203T, 32I**	11	Second	5 (45.5)	5 (45.5)	1 (9)
203T, 222E	23	Second	16 (69)	6 (26)	1 (4)

Viruses are grouped according with amino acid signature changes described in Figures 2 and 3. Substitutions located within the signal peptide were not considered.

a Amino acid changes are described using as reference the sequence of A/California/07/09.

b Refers to <4-fold, 4-fold and >4-fold dilutions of antiserum against A/California/07/09. Viruses with >4-fold reduced antigenic reactivity are considered as “low reactors”. Compared with the vaccine strain antiserum, the titres with A/England/195/2009 antiserum were within one-fold of difference in most cases and all the pandemic viruses showed no reactivity against A/Brisbane/59/07 antiserum; therefore these results are not included in the analysis.

c Groups where >50% of the viruses shows ≥4-fold reduced reactivity are highlighted in bold letter.

**Figure 5:**
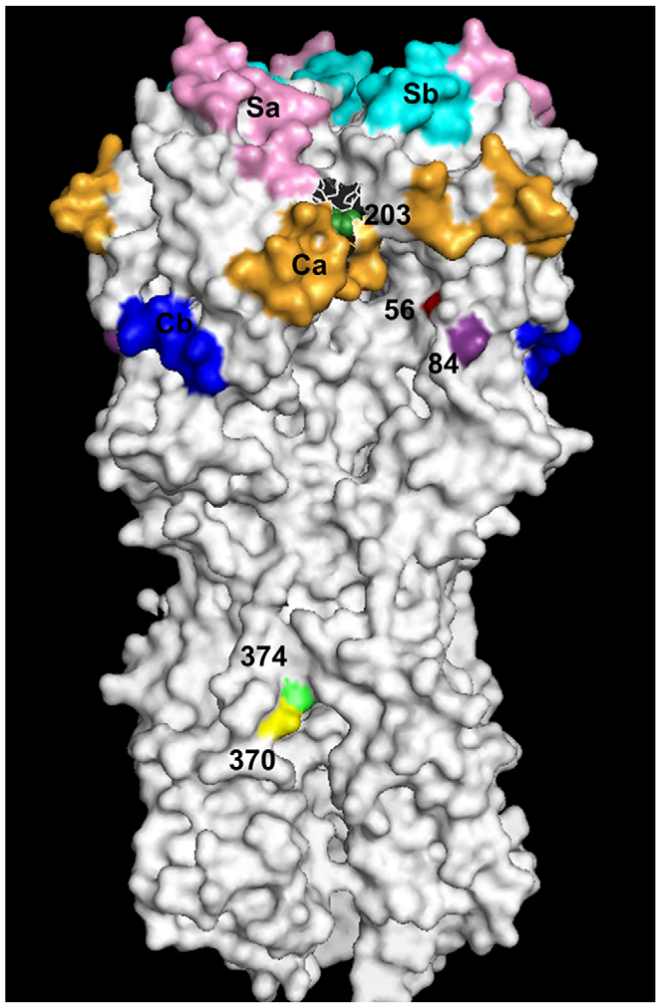
Surface representation of the hemagglutinin hetero-trimer from a pandemic (H1N1) 2009 strain [**17**]. Antigenic sites Ca (Orange), Cb (Blue), Sa (Pink) and Sb (Cyan) and residues of interest (56A [seq:73] (red), 84A [seq:101] (purple), 203A [seq:220) (dark green), 370B (HA2 43) [seq:387] (yellow) and 374B (HA2 47) [seq:391] (light green) are highlighted. Residue 203 is in a buried position only visible when residues 218, 238 & 239 are removed.

To further assess the possible significance of these substitutions associated with variation in the antigenic reactivity, we mapped them onto a surface representation of the HA trimer from a pandemic H1N1 (2009) representative strain (A/Darwin/2001/2009, PDB: 3M6S) [17] with highlighted antigenic sites, as described by Xu, Ekiert et al [15]. Residue 203T is located in antigenic site Ca although does not contribute to the surface of the molecule, being unclear whether it has any effect on the antigenic structure. Although a linear representation of the HA protein sequence shows that 56D and 84N are flanking antigenic site Cb (not shown), Figure 5 reveals that both changes are located in close proximity to antigenic sites Ca and Cb. Changes 370H and 374K, however, are located in the stalk region, far from the four antigenic sites and therefore do not seem to significantly affect their structure. Glycosylation can also mask protein epitopes to evade immune recognition. Although some of these changes also involved the replacement or the addition of asparagine (N), none were related to the acquisition of additional potential N-glycosylation sites or their loss.

## Discussion

Since the first cases of pandemic influenza (H1N1) 2009 infections were detected in late April until December 2009, covering the first two waves of the pandemic, there were 784,000 estimated clinical cases of pandemic influenza in the UK [8]. The estimation of number of cases in the second wave was almost twice that in the first wave (502,000 vs. 285,500)[18]. A peculiarity of the circulation of the pandemic virus within the UK is that it caused widespread transmission during the summer months of 2009 (first wave), despite extensive use of antiviral prophylaxis. In contrast, virtually all European countries experienced only a sporadic spread or small, local outbreaks over this period [6], [7]. Analysis of HA sequence diversity over the first two waves indicated that UK strains were closely related genetically to the current vaccine strain A/California/07/2009, with no obvious segregation of strains by geographical location, as observed by others [19]. Earlier strains were genetically more similar to the prototype than later strains, an observation consistent with the temporal accumulation of amino acid changes in the HA [20]. Overall genetic diversity among pandemic viruses was less than typically seen among seasonal influenza [21], [22], which can be attributed to a shorter evolutionary history since divergence, as the time of most recent common ancestor (TMRCA) for the pandemic virus was estimated around January 2009 [3], [23], [24]. The observed rate of substitution is similar to that estimated by Rambaut et al. [24], although higher than other estimations [3], [25]. This may be a sign of virus adaptation to the human host over our (longer) period of study.

The majority of substitutions characterising specific clusters in the tree are contained in no more than a dozen sites; some have been also reported by others [26], [27], [28]. We observed that, while some substitutions are plausibly the result of founder effects or early fixation, other polymorphisms coexist for extended periods, indicating the co-circulation of different variants. Widespread circulation of multiple viral strains in geographically distant places has been observed, confirming that global viral migration constituted a key element in the spread of the pandemic, as also seen for seasonal influenza [29].

In our study, 30% of 2009 UK viruses exhibited four-fold reduced reactivity to the vaccine strain antiserum compared with 6% for seasonal H1N1 influenza in 2007–2008. There are limitations in comparing the seasonal H1N1 influenza subtype which has circulated extensively in humans with the pandemic (H1N1) 2009 which has just emerged from an animal host as other factors could account for this variation in addition to immune selection. We emphasize that only a small number (3%) of viruses with greater than four-fold reduction (low reactors) were detected which were distributed throughout the phylogenetic tree, thus representing different lineages and enforcing the sporadic nature of their emergence. These are the viruses from which we might expect an expansion of lineage for the emergence of the next antigenically drifted variant. Despite 30% of viruses with four-fold reduction, no antigenic drift was detected during the second wave of 2009 and the vast majority of pandemic viruses were still considered as antigenically similar to the vaccine strain. The limited antigenic divergence seen in pandemic viruses from 2009 did not lead to antigenic drift during the subsequent influenza season (third wave) in 2010–2011 ([30], HPA unpublished data), therefore the recommended vaccine strain for H1N1 (2009) subtype is still A/California/07/09 for the next winter in the Northern hemisphere [31].

Most viruses described in Table 1 were isolated during the second wave and exhibited substitution S203T, which has potential to contribute to antigenic drift as it is located within antigenic site Ca, although its buried position leaves uncertainty about its effects [15], [32]. It is possible that this substitution affects the antigenic structure of the HA sufficiently to be responsible for the 30% of four-fold antigenic divergence. Given that there are few viruses without 203T to compare with, it is not possible to deduce whether 203T is necessary and sufficient to produce antigenic drift and additional studies should be conducted. It is also unclear whether the predominance of 203T could be attributed to immune selection or it is a consequence of further adaptive changes to the human host or optimisation of viral fitness. Factors supporting the latter include the fact that this change characterizes all viruses circulating in the UK (and elsewhere) from July onwards and has been highlighted as one of five substitutions across the genome of H1N1 (2009) strains that are concomitantly present in all recent pandemic viruses, characterising a new genetic variant that has replaced the viruses originally circulating in April 2009 (Baillie, Galiano et al, unpublished observations; [33]). Change 203T is still present in all UK HA sequences from the winter of 2010–2011 (third wave) [34]. The fixation of this change has occurred too quickly to be attributed to immune selection. Even when a substantial proportion of older adults have shown to have pre-existing immunity against the H1N1 (2009) virus [9], [35] and could be considered as a potential source of immune pressure during the early spread of the pandemic, the highest rates of infection were by far observed in school-age children and young adults (Figure S1 and [8]), therefore the population affected by the time 203T became fixed was largely naïve. On the other hand, substitutions at position 203 have not been previously described as potentially adaptive mutations, with the caveat that most studies have used avian influenza sequences [36], [37], [38].

Although most cluster-related signature changes were observed during the second wave, when we may expect to have more pressure for immune-driven changes as a significant proportion of the population will have been infected, the great majority were located outside any known antigenic site. Some of them were shown to be under positive selection, such as those at positions 84, 222 and 374. Substitution N84S (also referred as N101S) has been highlighted as fixed amongst H1N1 (2009) strains compared to previous seasonal H1N1 influenza, where less than 1% of viruses had 84S [39]. Reversion to the “seasonal” residue S84N may be a sign of further adaptation to the human host. Moreover, 75% of UK pandemic viruses with reversion to the “seasonal” residue S84N have a trend to lower antigenic reactivity. Substitution D222G has been observed to correlate with cases of severe or fatal disease. We found an increased frequency of substitution 222G in pandemic viruses isolated from fatal cases compared to viruses from non fatal cases. The difference between these groups was statistically significant, as also reported by others [12], [13], [14]. Substitutions at position 222 were previously shown to induce alterations in the receptor binding site [40]. Recent studies revealed that 222G variants bind a broader range of α2-3-linked sialyl receptors expressed on ciliated bronchial epithelial cells and on epithelia within the lung and suggested that these features of 222G mutants may be responsible for the exacerbation of disease [41], [42], but the exact relationship between 222G and case fatality is undetermined, since it appears neither necessary or sufficient for case fatality. Substitution E374K has been increasingly detected in up to 51% of UK viruses, in more than 80% of viruses from Singapore and in up to 40% of global isolates from December 2009 [33]. It has been identified as part of a highly conserved epitope in the 1918 H1N1 virus with a possible role in membrane fusion [43]. Changes in the antigenicity of this epitope would not necessarily be reflected in HI assays. The majority of viruses with changes 84N, 56D and 370H present (as well as 203T) showed four-fold or greater reduction in their antigenic reactivities. Unexpectedly, none of them was located within known antigenic sites, but there were no other relevant amino acid substitutions that could explain this trend. Structural analysis showed that 56D and 84N are located close to antigenic sites Ca and Cb. Although 370H is located in the stem region of the HA, it also form part of the same epitope that includes 374K, as mentioned above [43]. Some of these substitutions involve a change in polarity which may alter the distance to nearby amino acids that form part of the Ca site and thus indirectly alter the epitope conformation.

In summary, extensive analysis of the HA sequences from UK pandemic viruses isolated during 2009 revealed limited genetic diversity when compared to the circulation of seasonal influenza A, consistent with a shorter evolutionary time. Co-circulation of variants distinguished by specific amino acid substitutions was seen across a wide geographic spread. No antigenic drift occurred during 2009 as viruses considered as “low reactors” were detected in a low, non-significant proportion (3%). This conclusion is consistent with previous observations showing that, for seasonal influenza A, antigenic drift does not usually occur during a single epidemic season [44]. This is also a clear example that the cause-effect relationship between amino acid substitutions in the HA protein and the antigenic profile of influenza viruses is the result of a complex biological interaction, as also seen by antigenic mapping [22]. Whilst there is increasing recognition of the utility of sequence analysis for surveillance, development of antigenic profiles based solely on amino acid changes and their structural location within antigenic sites is not yet possible. This study highlights the caveats for analysis of diversity based only on sequence analysis and emphasises the need for phenotypic or antigenic characterisation.

## Materials and Methods

### Ethics Statement

Samples were taken during routine diagnostic work up by the hospital or community physicians. Only anonymised patient data (regional location, age, sample collection date) were used for this analysis. This was an observational study undertaken as part of pandemic surveillance. It was carried out under UK legislation NHS Act 2006 (section 251), which provides statutory support for disclosure of data by the NHS, and their processing by the Health Protection Agency (HPA) for communicable disease control. Written informed consent and explicit ethical approval was not sought for this reason. Health Protection Scotland is also embedded as part of the NHS in which the sharing of outbreak and investigation data is undertaken as part of their role in the coordination of national outbreaks.

### Influenza viruses used in this study

Respiratory samples were submitted to the Respiratory Virus Unit, Centre for Infections (CfI), HPA as part of influenza surveillance in the UK. Specimens detected as positive for pandemic influenza (H1N1) 2009 virus by laboratories in the Regional Microbiology Network (RMN) were sent to RVU for confirmation and virological investigations. These include specimens from patients hospitalised with influenza and community samples taken by general practitioners (GPs). Community samples were also obtained via the existing Royal College of General Practitioners (RCGP) influenza surveillance scheme for seasonal influenza which was extended into the summer months of 2009 since the outbreak of pandemic H1N1 at the end of the 2008/09 influenza season. Additional respiratory samples were also received as part of pandemic flu surveillance through a community based self-sampling scheme for pandemic influenza (NHS Direct/NPFS for National Pandemic Flu Service) established in England from May 2009 to February 2010.

Combined nose and throat swab specimens were analysed at CfI by real-time RT-PCR for detection of influenza A, and subtyped for seasonal H1, H3, and pandemic (H1N1) 2009 viruses [45], [46], [47], [48]. Prior to June 2009, pandemic H1N1 virus was diagnosed by sequencing of the influenza A PCR amplicon [45], and from June onwards by a swine lineage N1 real-time PCR confirmatory assay [46]. For non-sentinel samples from hospitalized patients, prior to June influenza A unsubtypable specimens were confirmed at CfI for pandemic (H1N1) 2009 virus; after June 2009, confirmatory testing of non-sentinel samples was performed in the referring laboratories.

### Influenza virus isolation and antigenic characterisation

Viruses were isolated from clinical specimens either in Madin-Darby canine kidney cells (MDCK) or the SIAT1 derivative [49] in the presence of 1.25 µg/ml of trypsin and stored at −80°C. A haemagglutination inhibition (HI) assay was used for antigenic characterisation of virus isolates using a standard method. Briefly, post-infection ferret sera to the influenza virus reference strains A/California/07/2009 (vaccine strain), A/England/195/2009 (UK reference strain) and A/Brisbane/59/07 (the previous seasonal H1N1 vaccine strain) were treated with receptor-destroying enzyme (RDE) (Denka Seiken, Japan) to remove non-specific inhibitors. Reference viruses and virus isolates were standardised to 8HAU and incubated with serial dilutions of ferret sera. The HI titre was defined as the reciprocal of the highest dilution of serum which still inhibited virus agglutination of turkey red blood cells. Virus isolate HI titres were compared to reference virus homologous HI titres to determine reactivity.

### Sequencing of full length HA

685 viruses were chosen for sequencing of the complete coding region of the HA gene. Virus RNA was extracted from either aliquots of original respiratory material or from viral isolates using either the QIAxtractor robot (Qiagen Ltd, West Sussex, England) or the NucliSENS easyMag system (bioMérieux UK Ltd, Hampshire, England). Reverse transcription was performed using Superscript III RT reverse transcriptase (Invitrogen Ltd, Paisley, England) following manufacturer's instructions and the universal primer for influenza Uni12 [50]. The complete coding region of the HA gene was then amplified using Platinum Pfx, a proof-reading DNA polymerase (Invitrogen). Primers for amplification and sequencing were supplied by Eurofins MWG Operon (Ebersberg, Germany) and are available upon request. RT-PCR products were prepared for sequencing by purifying with Ampure magnetic beads on the Biomek NxP robot (Beckman Coulter, High Wycombe, England).The cleaned PCR products were used for sequencing with the ABI Big Dye Terminator Kit v3.1 (Applied Biosystems) followed by clean-up of products with the CleanSeq magnetic beads on the Biomek NxP robot (Beckman Coulter). Automated sequence detection was performed on a 48-capillary ABI 3730 Genetic Analyser (Applied Biosystems, Warrington, UK). PCR clean up and sequencing reactions were performed by the Genomic Services Unit at the Department for Bioanalysis and Horizon Technologies, CfI. Raw sequencing data was edited and assembled into contigs using Sequencher 4.9 [51]. Genbank accession numbers are listed in Supplementary Information (Dataset S1).

For the integrity and consistency of data and updates, sequences were deposited in a custom PostgreSQL database designed in-house. Ease and consistency of access was achieved via a custom in-house web application (HPA Influenza Isolates database http://www.hpa-bioinformatics.org.uk/influenza_isolates), and integrated with epidemiological data via an interface with BioNumerics 5.10 [52] and LIMS software.

#### Sequence accession numbers

All the sequences were submitted to GenBank. Accession numbers are detailed in Dataset S1.

### Phylogenetic analyses

Alignments were prepared and edited as above. A phylogeny was reconstructed with Bayesian inference via MrBayes [53], with an appropriate substitution model chosen via jModeltest [54]. Convergence of the Markov chain was judged by the sum of split frequencies, PSRF (potential scale reduction factor) and visual inspection of the likelihood plot. One-hundred samples were collected and a consensus tree constructed. This and other trees were visualized with Dendroscope V2.3 [55]. BEAST [56] was used with a relaxed model of molecular evolution to calculate divergence times and thus the estimated rate of substitution at every node. These were used to calculate an overall median rate of substitution.

Potential N-glycosylation sites in the deduced amino acid sequences were predicted using NetNGlyc version 1.0 [57]. Only sites featuring the sequence Asn-Xaa-Ser/Thr without Proline at Xaa and a potential score above the threshold of 0.5 were considered as predicted glycosylated sites.

The structural image of the HA trimer was created using PyMOL version 1.1 [58]. The California/2007/HA amino acid sequence was aligned separately against the A and B chains of the 3M6S structure [17], allowing amino numbering to be mapped directly from sequence to structure. Chains G to L were excised from the structure of 3M6S to leave a single, 6 chain, representation of the HA structure. The molecular surface of the HA was calculated using a probe of 1.4 Angstroms radius and the residues corresponding to the antigenic sites and residues of interest, identified in the alignment, were coloured as described.

### Analysis of selective pressures

Selection pressures were evaluated via the Datamonkey suite [59]. The evolutionary model for analyses - HKY85 - was selected with jModeltest and HyPhy [60]. Due to an excess of faint selection signals, the relatively small amount of evolutionary time and to avoid possible methodological problems, several approaches were used to assess selection: Single Likelihood Ancestral Counting (SLAC), Fixed-Effects Likelihood (FEL) and Random Effects Likelihood (REL) methods. Results were classified as having good significance (i.e. a p-value<0.1 or a Bayes factor >10) or strong significance (i.e. a p<0.01 or a Bayes factor >100). Given the limitations of the data and the possibility of false positives, the pool of possible sites was winnowed down to those that had demonstrated at least 1 strongly significant result, or two results of good significance. Two subsets were tested, using the sequences collected from the first and second waves of pandemic 2009.

## Supporting Information

Figure S1 **Lab-confirmed cases vs sequenced cases by age group (A) and geographical region (B).** The highest proportion of lab-confirmed cases in the 5–14 age group is coincident with the highest rate of consultation and also with the highest seroincidence in this age group during the first and second wave of pandemic in the UK [8], [9].
(TIF)

Figure S2 **Phylogeny of sequenced UK HA sequences.** Evolutionary tree constructed by MrBayes and coloured according to first or second wave viruses. Nodes labelled with posterior probabilities. A/California/07/2009 used as outgroup. Antigenically low reactor viruses are boxed and labelled.
(TIF)

Figure S3 **Amino acid positions of HA from pandemic influenza (H1N1) 2009 under positive or negative selection.** Three different methods within the DataMonkey suite were used: Single Likelihood Ancestral Counting (SLAC), Fixed-Effects Likelihood (FEL) and Random Effects Likelihood (REL) methods. A consensus approach was used where only those sites with strong evidence from one or more methods or good evidence from two or more methods were listed. The magnitude of selection is given as dN-dS as calculated by REL.
(TIF)

Dataset S1 **Genbank accession numbers of UK sequences used in this study.**
(DOC)
