# Supplementary figures and images for: Evolutionary Pathways of the Pandemic Influenza A (H1N1) 2009 in the UK
Source: PLoS One. 2011 Aug 24;6(8):e23779. doi: 10.1371/journal.pone.0023779 (PMC3161082; doi:10.1371/journal.pone.0023779)

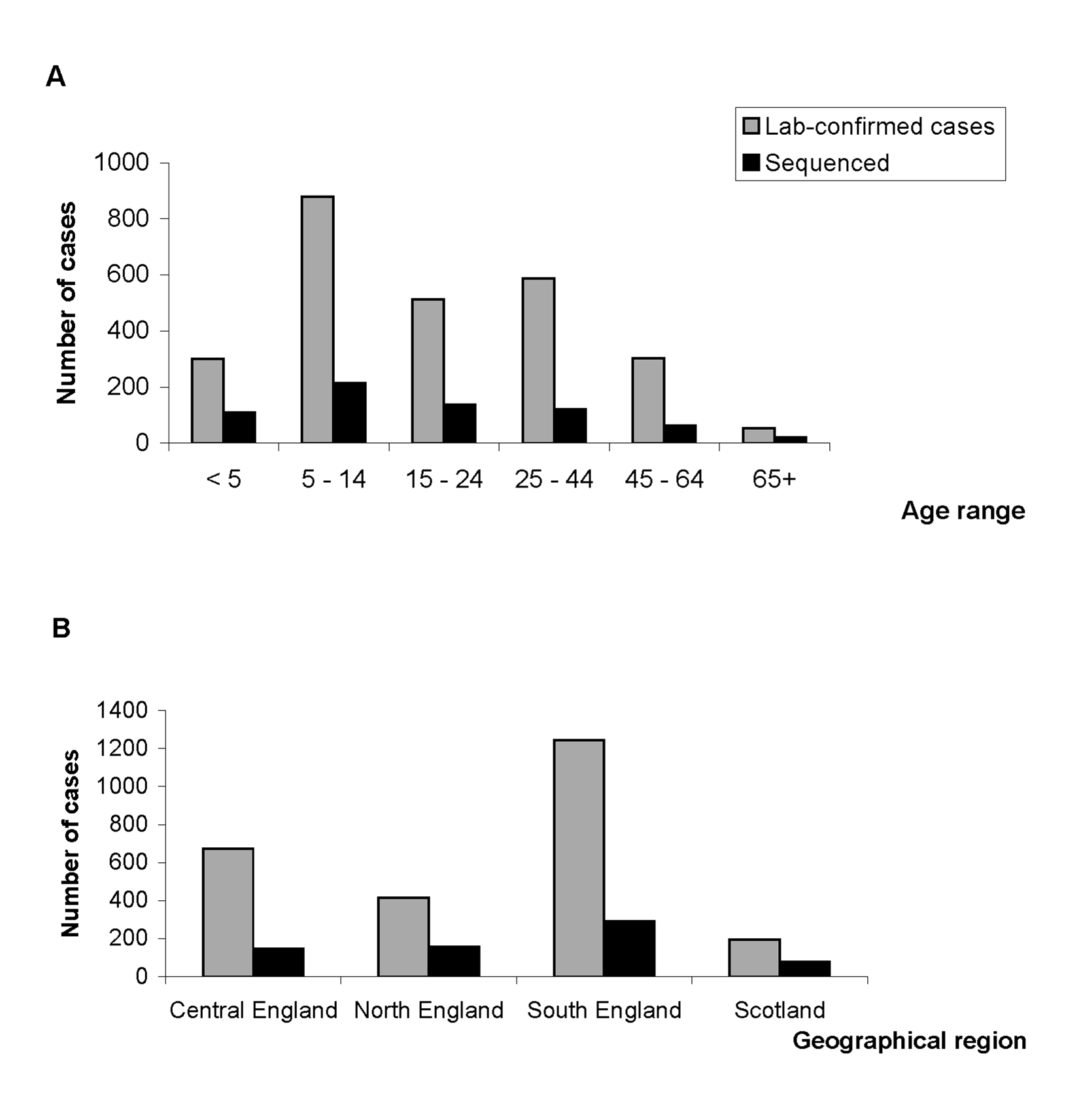

Supplement: Figure S1 — Lab-confirmed cases vs sequenced cases by age group (A) and geographical region (B). The highest proportion of lab-confirmed cases in the 5–14 age group is coincident with the highest rate of consultation and also with the highest seroincidence in this age group during the first and second wave of pandemic in the UK [8], [9]. (TIF) [file pone.0023779.s001.tif]

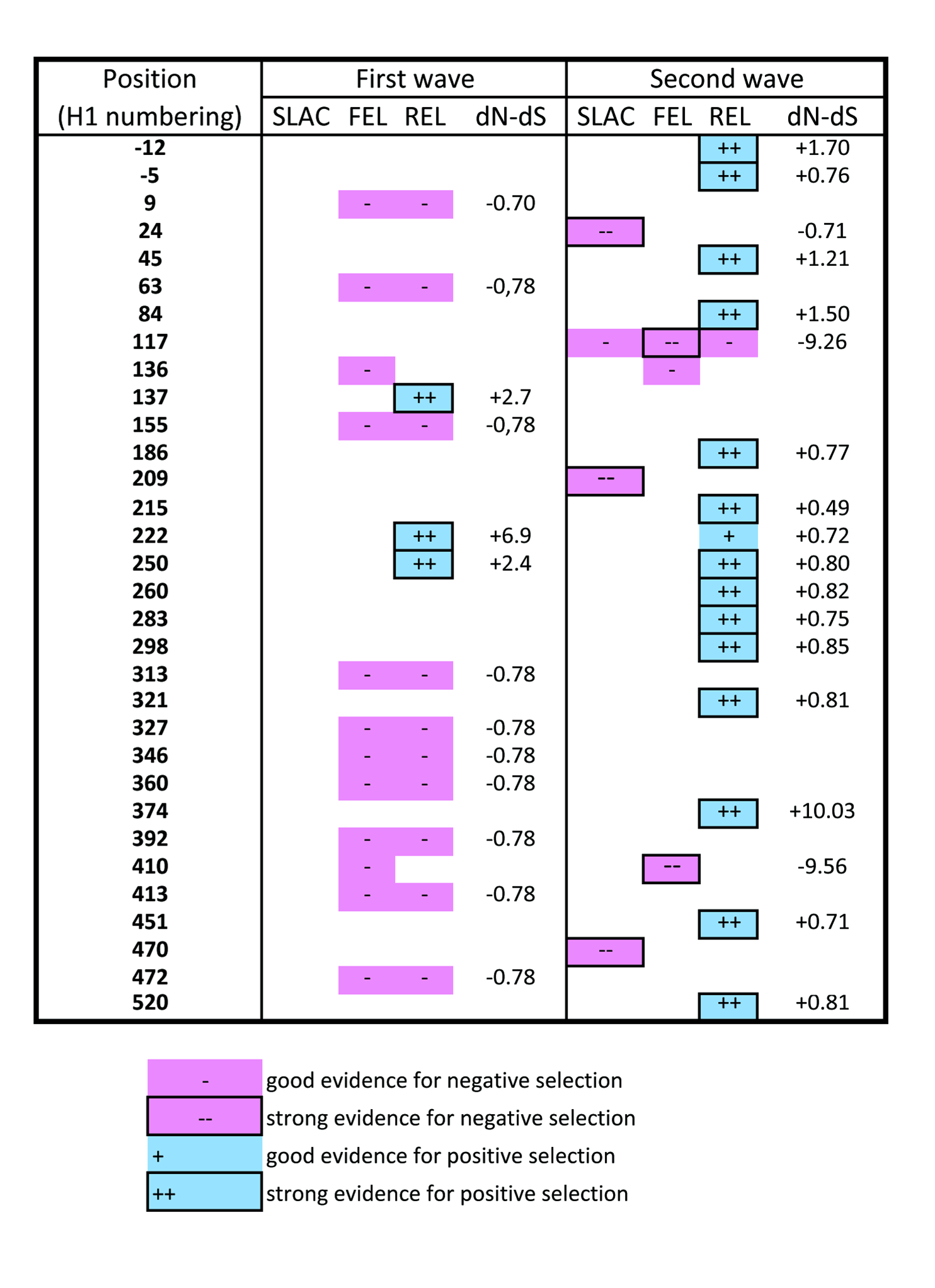

Supplement: Figure S3 — Amino acid positions of HA from pandemic influenza (H1N1) 2009 under positive or negative selection. Three different methods within the DataMonkey suite were used: Single Likelihood Ancestral Counting (SLAC), Fixed-Effects Likelihood (FEL) and Random Effects Likelihood (REL) methods. A consensus approach was used where only those sites with strong evidence from one or more methods or good evidence from two or more methods were listed. The magnitude of selection is given as dN-dS as calculated by REL. (TIF) [file pone.0023779.s003.tif]
