# Supplementary material for: Evolutionary Pathways of the Pandemic Influenza A (H1N1) 2009 in the UK
Source: PLoS One. 2011 Aug 24;6(8):e23779. doi: 10.1371/journal.pone.0023779 (PMC3161082; doi:10.1371/journal.pone.0023779)
Supplement: Dataset S1 — Genbank accession numbers of UK sequences used in this study. (DOC) [file pone.0023779.s004.doc]

**Genbank accession numbers of UK sequences used in this study:**

CY070157-CY070746 and: CY065142,CY065150,CY065158,CY065166,CY065182,CY065190,CY065198,CY065206,CY065214,CY065222,CY065230,CY065238,CY065254,CY065262,CY065270,CY065278,CY065286,CY065302,CY065310,CY065318,CY065326,CY065334,CY065342,CY065350,CY065358,CY065366,CY065374,CY065382,CY065390,CY065398,CY065406,CY065414,CY065422,CY065430,CY065438,CY065446,CY065454,CY065462,CY065470,CY065478,CY065486,CY065494,CY065502,CY065510,CY065518,CY065526,CY065534,CY065542,CY065550,CY065558,CY065566,CY065574,CY065582,CY065590,CY065598,CY065606,CY065614,CY065622,CY065630,CY065638,CY065646,CY065654,CY065662,CY065670,CY065686,CY065694,CY065702,CY065710,CY065718,CY065726,CY065734,CY065742,CY069749,CY069757,CY069773,CY069781,CY069789,CY069813,CY069853,CY069869,CY069877,CY069885,CY069893,CY069901,CQ166657,HM567712,HM567960,HM567984,HM567992,HM568000,HM568032,HM568040,HM568096,HM568120,HM568128
